# Pre‐Implantation Balloon Aortic Valvuloplasty and Clinical Outcomes Following Transcatheter Aortic Valve Implantation: A Propensity Score Analysis of the UK Registry

**DOI:** 10.1161/JAHA.116.004695

**Published:** 2017-02-18

**Authors:** Glen P. Martin, Matthew Sperrin, Rodrigo Bagur, Mark A. de Belder, Iain Buchan, Mark Gunning, Peter F. Ludman, Mamas A. Mamas

**Affiliations:** ^1^ Health e‐Research Centre University of Manchester Manchester United Kingdom; ^2^ Division of Cardiology Department of Medicine London Health Sciences Centre University Hospital Western University London Ontario Canada; ^3^ James Cook University Hospital Middlesbrough United Kingdom; ^4^ Royal Stoke Hospital University Hospitals North Midlands Stoke‐on‐Trent United Kingdom; ^5^ Queen Elizabeth Hospital Birmingham United Kingdom; ^6^ Keele Cardiovascular Research Group Keele University Stoke‐on‐Trent United Kingdom

**Keywords:** aortic stenosis, balloon valvuloplasty, balloon‐expandable, self‐expandable, transcatheter aortic valve implantation, Aortic Valve Replacement/Transcather Aortic Valve Implantation

## Abstract

**Background:**

Aortic valve predilation with balloon aortic valvuloplasty (BAV) is recommended before transcatheter aortic valve implantation (TAVI), despite limited data around the requirement of this preprocedural step and the potential risks of embolization. This study aimed to investigate the trends in practice and associations of BAV on short‐term outcomes in the UK TAVI registry.

**Methods and Results:**

Eleven clinical endpoints were investigated, including 30‐day mortality, myocardial infarction, aortic regurgitation, valve dysfunction, and composite early safety. All endpoints were defined as per the VARC‐2 definitions. Odd ratios of each endpoint were estimated using logistic regression, with data analyzed in balloon‐ and self‐expandable valve subgroups. Propensity scores were calculated using patient demographics and procedural variables, which were included in the models of each endpoint to adjust for measured confounding. Between 2007 and 2014, 5887 patients met the study inclusion criteria, 1421 (24.1%) of whom had no BAV before TAVI valve deployment. We observed heterogeneity in the use of BAV nationally, both temporally and by center experience; rates of BAV in pre‐TAVI workup varied between 30% and 97% across TAVI centers. All endpoints were similar between treatment groups in SAPIEN (Edwards Lifesciences Inc., Irvine, CA) valve patients. After correction for multiple testing, none of the endpoints in CoreValve (Medtronic, Minneapolis, MN) patients were significantly different between patients with or without predilation.

**Conclusions:**

Performing TAVI without predilation was not associated with adverse short‐term outcomes post procedure, especially when using a balloon‐expandable prosthesis. Randomized trials including different valve types are required to provide conclusive evidence regarding the utility of predilation before‐TAVI.

## Introduction

Transcatheter aortic valve implantation (TAVI) is an effective treatment option for multimorbid patients with severe symptomatic aortic stenosis who are either not suitable for conventional surgical aortic valve replacement or who are deemed high‐risk surgical candidates.^1^, ^2^, ^3^, ^4^

During the TAVI procedure, recommendations have included the use of balloon aortic valvuloplasty (BAV) to predilate the aortic valve before deployment of the transcatheter valve. Such predilation is intended to aid delivery of the prosthesis across the valve, enhance prosthesis expansion in the aortic annulus, provide information about the aortic annulus size, and, potentially, improve hemodynamic performance during the TAVI procedure.^5^ Additionally, BAV during TAVI can be used to evaluate possible coronary occlusion in patients with low coronary height. However, BAV is associated with complications, including stroke, conduction disturbances, and severe aortic regurgitation.^6^, ^7^ Thus, it is possible that the routine use of BAV in TAVI procedures actually increases procedural risk. Whilst it is routine for many TAVI centers to predilate using BAV, recent preliminary studies have indicated that TAVI without predilation is feasible in both Edwards SAPIEN and Medtronic CoreValve prostheses.^8^, ^9^, ^10^, ^11^, ^12^, ^13^, ^14^ However, much of the previously published data in this area are derived from small, single‐center studies and subject to sampling bias, with little data on utility of BAV and its associated clinical outcomes following TAVI in large, multicenter TAVI registries.

Therefore, this analysis was undertaken in the UK TAVI registry to investigate patterns of BAV use across the UK and its association with short‐term clinical outcomes following TAVI.

## Methods

### UK TAVI Registry

The UK TAVI registry uses a Web‐based interface provided by the National Institute of Cardiovascular Outcomes Research to collect data prospectively on every TAVI procedure conducted in the UK.^15^ There are currently 34 centers running active TAVI programs, with data collection being mandatory.^15^ The data set comprises 95 variables, detailing patient demographics, risk factors for intervention, procedural details, and adverse outcomes up to the time of hospital discharge. Patient life status was provided by record linkage with the Office for National Statistics for English and Welsh patients. Mortality information for Northern Irish patients and the majority of Scottish patients was unavailable, and, consequently, these patients were removed from the analysis.

This study analyzed data from January 2007 to December 2014. The Edwards SAPIEN (Edwards Lifesciences Inc., Irvine, CA) and the Medtronic CoreValve (Medtronic, Minneapolis, MN) prostheses were available to all centers throughout the study period.

### Study Design

Endpoints in this study were 30‐day mortality and the following events occurring up to hospital discharge: myocardial infarction (MI); stroke; paravalvular leak (PVL)/moderate‐severe aortic regurgitation (AR); coronary artery obstruction (CAO); valve dysfunction requiring repeat procedure; permanent pacemaker implantation requirement; device migration; kidney injury; major vascular complications (MVC); and composite early safety. All endpoints were defined as given in the VARC‐2 definitions.^16^

This analysis defined BAV procedures based on the timing of any such procedure relative to the time of TAVI. Specifically, we distinguished the following timings: (1) BAVs completed before the date of TAVI (Before‐TAVI BAV); (2) BAVs completed as part of the TAVI procedure, but before valve deployment (During‐TAVI BAV); and (3) no BAV before or during the TAVI procedure (Direct TAVI). Because the aim of this study was to investigate the effect of predilation on TAVI outcomes, the main analysis excluded any patient who had a BAV before the date of TAVI; hence, the main analysis compared endpoints across patients with a During‐TAVI BAV (but none before) and Direct TAVI, with the latter group taken as the reference. All patients with missing treatment group identifiers were excluded.

Additionally, to investigate whether the timing of the BAV relative to the TAVI procedure was associated with outcomes, we conducted a sensitivity analysis that did not exclude those patients who had a BAV before the date of TAVI. Hence, the sensitivity analysis compared outcomes across all 4 possible treatment groups: (1) Before‐TAVI BAV and During‐TAVI BAV; (2) Before‐TAVI BAV and No During‐TAVI BAV; (3) No Before‐TAVI BAV and During‐TAVI BAV; and (4) No Before‐TAVI BAV and No During‐TAVI BAV (Direct TAVI). Here, groups 3 and 4 comprised exactly those patients as in the main analysis.

Given that the effects of BAV on outcomes post‐TAVI were potentially dependent on the expansion method of the valve type (balloon‐ or self‐expandable), all analyses were completed in device‐specific subgroups (SAPIEN vs CoreValve). Patients were excluded only from the valve‐subgroup analyses if they were not treated with a SAPIEN or CoreValve prosthesis or if the valve type was unknown.

### Statistical Analysis

Continuous data were presented as means and SDs, with group comparisons done with ANOVA. Categorical data were presented as counts and percentages with group comparisons done using the chi‐squared test.

Every variable with missing data was imputed using multiple imputation.^17^ Ten imputed data sets were generated using multiple imputation by chained equations. The imputation model for each variable included the majority of other variables in the UK TAVI registry. Additionally, to avoid underestimation of covariate‐outcome associations, all endpoints were used in the imputation models for missing covariates.^18^ After such imputation, the imputed outcome values were returned to the original values (ie, missing), following the so‐called multiple imputation, then deletion approach.^19^ All patients with missing life status were excluded from the analysis; patients with other endpoints missing were only excluded from the analysis of that particular endpoint. Analyses were undertaken in each data set separately, before pooling results according to Rubin's rules.^17^

To investigate clinical outcomes across treatment groups, propensity scores (PSs) for being in each treatment group were calculated for all patients to control for potential confounders and baseline differences.^20^, ^21^ A logistic regression model calculated each patient's PS, given the baseline covariates, which included every variable listed in Table 1 (except the Logistic EuroSCORE [LES] and Society of Thoracic Surgeons Score for Prediction of Mortality [STS] score), in addition to a TAVI center experience indicator and year of procedure. For the sensitivity analysis, a multinomial logistic regression model was used to calculate each patient's PS for each treatment group, which included exactly the same covariates as for the main analysis. Odds ratios (ORs) for each endpoint across BAV treatment groups were estimated using a logistic regression model that was fitted to each outcome with the treatment group indicator and the PS as covariates. A Bonferroni correction was applied to account for multiple testing.

**Table 1:** Baseline Characteristics Across the Treatment Groups in the Main Analysis That Excluded BAVs Conducted Before TAVI

Variable	Whole Cohort (n=5887)	During‐TAVI BAV (n=4466)	Direct TAVI (n=1421)	*P* Value	Missing (%)
Age, mean (SD)	81.3 (7.5)	81.5 (7.2)	80.5 (8.2)	<0.001	0 (0.00)
Female, n (%)	2755 (46.8)	2125 (47.6)	630 (44.3)	0.03	21 (0.36)
Diabetic, n (%)	1351 (22.9)	1019 (22.8)	332 (23.4)	0.70	6 (0.10)
Smoker, n (%)	3051 (51.8)	2351 (52.6)	700 (49.3)	0.10	201 (3.4)
Creatinine, mean (SD)	113.7 (64.9)	112.8 (64.0)	116.3 (67.5)	0.08	44 (0.75)
Renal failure^a^, n (%)	351 (6.0)	250 (5.6)	101 (7.1)	0.05	72 (1.2)
Previous MI, n (%)	1246 (21.2)	936 (21.0)	310 (21.8)	0.50	6 (0.10)
Pulmonary disease, n (%)	1648 (28.0)	1262 (28.3)	386 (27.2)	0.38	51 (0.9)
Neurological disease, n (%)	1011 (17.2)	790 (17.7)	221 (15.6)	0.07	6 (0.10)
Extracardiac arteriopathy, n (%)	1390 (23.6)	1085 (24.3)	305 (21.5)	0.02	51 (0.87)
Calcification of ascending aorta, n (%)	1106 (18.8)	923 (20.7)	183 (12.9)	<0.001	44 (0.75)
Atrial fibrillation, n (%)	1434 (24.4)	1071 (24.0)	363 (25.5)	0.28	68 (1.2)
Previous cardiac surgery, n (%)	1884 (32.0)	1299 (29.1)	585 (41.2)	<0.001	6 (0.10)
Previous PCI, n (%)	1141 (19.4)	877 (19.6)	264 (18.6)	0.40	6 (0.10)
Height, mean (SD)	1.65 (0.10)	1.64 (0.10)	1.65 (0.10)	0.01	110 (1.9)
Weight, mean (SD)	74.1 (16.4)	73.9 (16.4)	74.8 (16.4)	0.06	87 (1.5)
CCS class 4, n (%)	70 (1.2)	53 (1.2)	17 (1.2)	0.99	10 (0.17)
NYHA ≥III, n (%)	4708 (80.0)	3642 (81.5)	1066 (75.0)	<0.001	15 (0.25)
Pulmonary hypertension, n (%)	677 (11.5)	499 (11.2)	178 (12.5)	0.001	1652 (28.1)
Aortic valve area, mean (SD)	0.68 (0.22)	0.66 (0.20)	0.74 (0.28)	<0.001	325 (5.5)
Aortic valve peak gradient, mean (SD)	75.6 (25.9)	78.4 (25.4)	66.3 (25.5)	<0.001	222 (3.8)
LVEF <50%, n (%)	2160 (36.7)	1628 (36.5)	532 (37.4)	0.47	28 (0.48)
One or more diseased vessels, n (%)	2507 (42.6)	1952 (43.7)	555 (39.1)	0.001	71 (1.2)
Left main stem disease, n (%)	271 (4.6)	215 (4.8)	56 (3.9)	0.22	102 (1.7)
Nonelective procedure, n (%)	702 (11.9)	478 (10.7)	224 (15.8)	<0.001	2 (0.03)
LES, mean (SD)^b^	21.4 to 21.5 (13.7–13.9)	21.4 to 21.5±13.4 to 13.5	22.8 to 23.2±14.8 to 15.1	<0.001	NA
STS score, mean (SD)^b^	4.9 to 5.0 (4.0–4.1)	5.0 to 5.0±3.7 to 3.8	5.1 to 5.2±4.6 to 4.7	0.01	NA
Access site					5 (0.08)
Transfemoral, n (%)	4385 (74.5)	3326 (74.5)	1059 (74.5)	0.92	
Transapical, n (%)	952 (16.2)	709 (15.9)	243 (17.1)	0.28	
Subclavian, n (%)	223 (3.8)	194 (4.3)	29 (2.0)	<0.001	
Other, n (%)	322 (5.5)	235 (5.3)	87 (6.1)	0.23	

BAV indicates balloon aortic valvuloplasty; CCS, Canadian Cardiovascular Society; LES, Logistic EuroSCORE; LVEF, left ventricular ejection fraction; MI, myocardial infarction; NA, not applicable; NYHA, New York Heart Association; PCI, percutaneous coronary intervention; STS, Society of Thoracic Surgeons Score for Prediction of Mortality; TAVI, transcatheter aortic valve implantation.

a Defined as creatinine >200 μmol/L or dialysis for renal failure.

b The Logistic EuroSCORE and STS models were calculated using the imputed data, and so ranges are given for these variables for the summary measures across the 10 multiply imputed data sets; variables that were included in either model, but were not recorded in the UK TAVI registry, were assumed risk‐factor absent.

Patient characteristics that resulted in a higher probability to perform predilation were identified by deriving a logistic regression model with During‐TAVI BAV (no before BAV) as the dependent variable. Predictors associated with the use of predilation were investigated by backward selection using Akaike information criterion in each imputed data set, resulting in 10 (potentially different) sets of selected predictors. Predictors that were selected in more than 50% of the 10 imputed data sets were identified as independent predictors of During‐TAVI BAV, following the so‐called majority method of selecting variables in multiple imputed data.^22^ Given the selected predictors, a logistic regression model was fitted in each of the 10 imputed data sets with estimated coefficients and SEs, then pooled according to Rubin's rules.^17^

R (version 3.3.1; R Foundation for Statistical Computing, Vienna, Austria)^23^ was used for all statistical analyses. Graphical plots where made using the ggplot2 package,^24^ and the mice package was used for the multiple imputation.^25^

## Results

**Figure 1:**
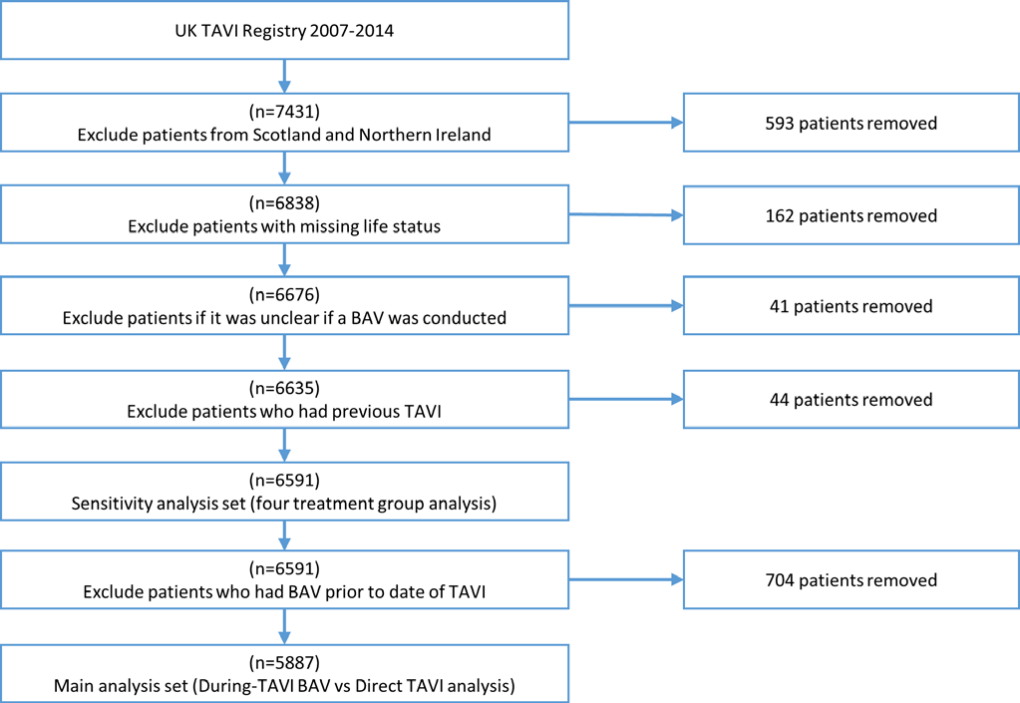
Flow chart illustrating the exclusion criteria applied to the UK TAVI registry. BAV indicates balloon aortic valvuloplasty; TAVI, transcatheter aortic valve implantation.

From January 2007 to December 2014, 7431 patients underwent a TAVI procedure in the UK. The flow of patients through the steps of exclusion criteria is illustrated in Figure 1. Specifically, the analysis set for the main analysis comprised of 5887 patients; 1421 patients (24.1%) had no BAV (Direct TAVI) and 4466 patients (75.9%) had a During‐TAVI BAV. Together, 3201 patients had a SAPIEN valve, 2467 had a CoreValve, and the remaining 219 were treated with another or unknown valve type. For the sensitivity analysis, which did not exclude Before‐TAVI BAV patients, the analysis set included exactly those patients in the main analysis in addition to 507 patients who had a Before‐ and During‐TAVI BAV and 197 who had a Before‐TAVI BAV but no During‐TAVI BAV.

Summary statistics of baseline characteristics for the main analysis are given in Table 1. The During‐TAVI BAV group had significantly higher mean age and higher proportions of patients with extracardiac arteriopathy, calcification of ascending aorta, New York Heart Association (NYHA) class III or IV, and 1 or more disease coronary vessels, but significantly smaller proportions of patients with previous cardiac surgery and pulmonary hypertension. Patients in the During‐TAVI BAV group had a significantly smaller mean aortic valve area and significantly larger aortic peak gradient than in the Direct TAVI group (*P*<0.001), although the proportion of patients with impaired left ventricular function at the time of the TAVI procedure was similar (*P*=0.47). The LES and STS score models were calculated in each multiply imputed data set using the variables and coefficients previously published.^26^, ^27^ Hence, the ranges of the mean and SDs across each imputed data set are given; predicted risk as estimated by both models was significantly different across treatment groups (Table 1).

### Trends in BAV Practice

**Figure 2:**
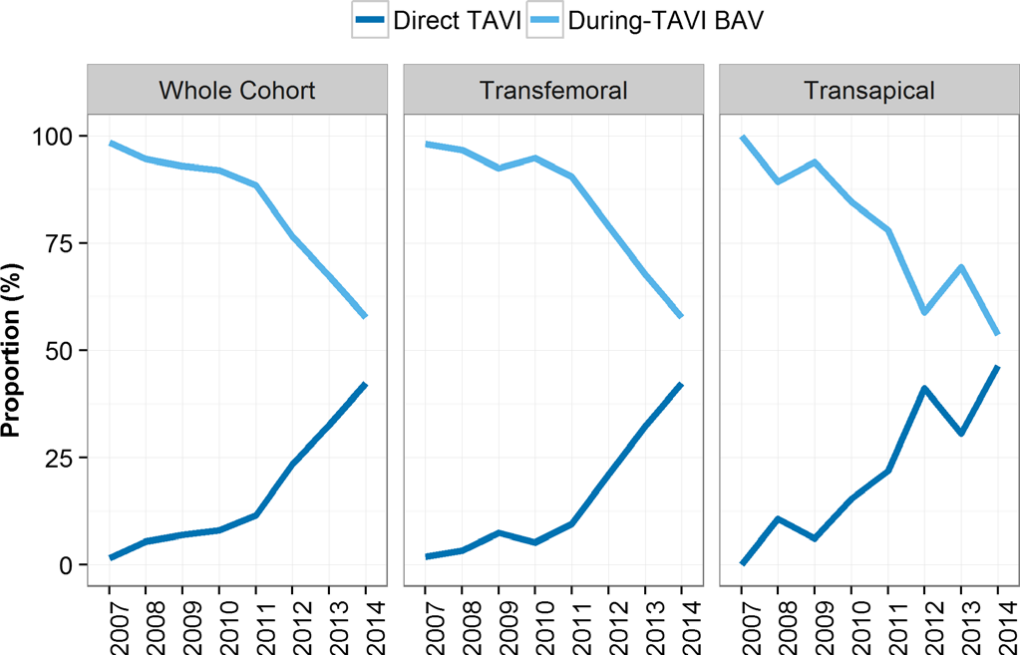
Longitudinal changes in the proportion of TAVI patients having BAV during‐TAVI BAV (no BAV before TAVI) and direct TAVI in the whole cohort and by access route. BAV indicates balloon aortic valvuloplasty; TAVI, transcatheter aortic valve implantation.

**Figure 3:**
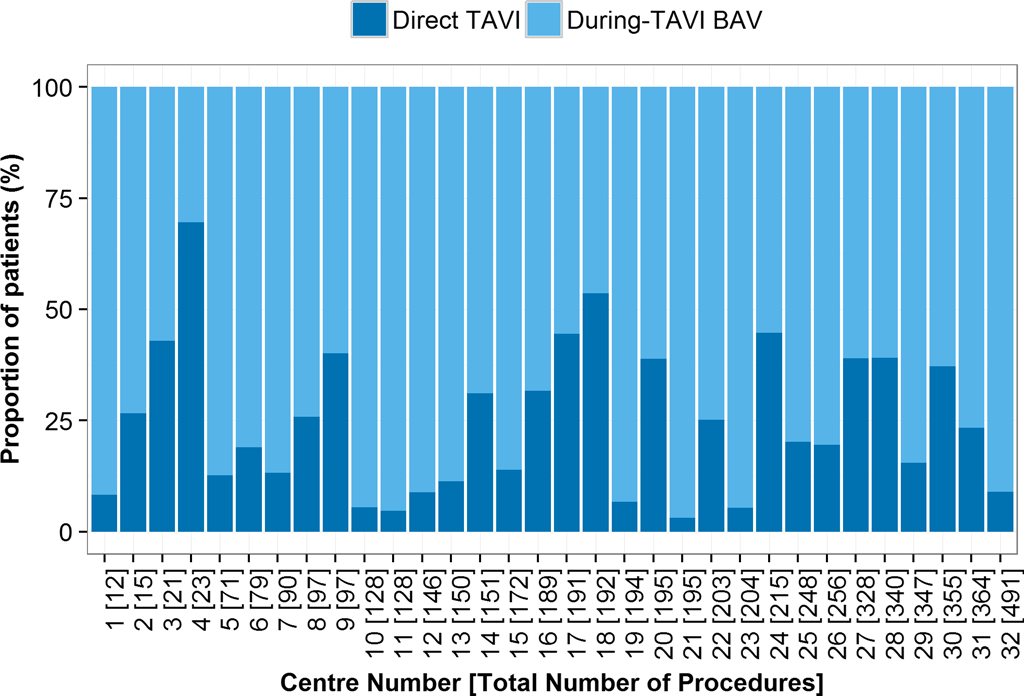
Proportion of patients having during‐TAVI BAV (no BAV prior to TAVI) and direct TAVI over the 32 centers running active TAVI programs in England and Wales by 2014. The centers on the *x*‐axis have been sorted based on the total number of TAVI procedures each has conducted. BAV indicates balloon aortic valvuloplasty; TAVI, transcatheter aortic valve implantation.

**Figure 4:**
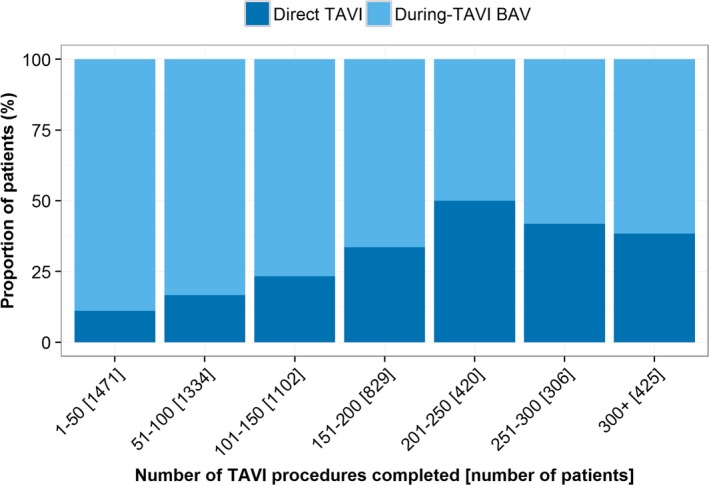
Proportion of patients in each treatment group by center experience. The *x*‐axis shows the number of TAVI procedures conducted within a center before each patient within that center. BAV indicates balloon aortic valvuloplasty; TAVI, transcatheter aortic valve implantation.

Between 2007 and 2014, there was a decreasing trend in the proportion of patients undergoing predilation in the whole cohort (*P*<0.001) and by access route (*P*=0.001) (Figure 2). A similar pattern of longitudinal behavior was observed over SAPIEN and CoreValve patients. Additionally, there was heterogeneity in practice among centers, with During‐TAVI BAV group rates varying from 30% to 97% (Figure 3). Interestingly, there was a visual trend of decreased use of BAV for successive increases in center experience, with the exception of the 2 very highest‐volume groups (251–300 and 300+), which represented just 7 centers (Figure 4). Specifically, when a center had undertaken between 1 and 50 previous TAVI procedures, rates of During‐TAVI BAV were 89%, but this had decreased to 50% when centers had undertaken between 201 and 250 previous TAVIs.

### TAVI Outcomes by BAV Treatment Group

Table 2 gives the PS adjusted ORs for each outcome in the whole cohort for the main analysis. Before adjusting for multiple testing, patients with a During‐TAVI BAV had increased odds of having a permanent pacemaker (OR of 1.30). However, this was not significant after correcting for multiplicity (Table 2). There were no other significant differences in other endpoints between the 2 treatment groups. Similar findings were obtained in the sensitivity analysis of the 4 treatment groups (Table 3).

**Table 2:** Crude Event Rates and PS Regression Adjusted ORs for Each of the Considered Outcomes in the Whole Cohort for the Main Analysis That Excluded BAVs Conducted Before TAVI

Outcome	During‐TAVI BAV (n=4466)	Direct TAVI (n=1421)	PS‐Adjusted OR (95% CI) Without Bonferroni Correction	PS‐Adjusted OR (95% CI) With Bonferroni Correction
30‐day mortality	239/4466 (5.4%)	63/1421 (4.4%)	1.04 (0.76, 1.42)	1.04 (0.63, 1.72)
MI	36/4442 (0.81%)	8/1411 (0.57%)	1.03 (0.45, 2.35)	1.03 (0.27, 3.93)
Stroke	132/4445 (3.0%)	35/1409 (2.5%)	0.91 (0.60, 1.37)	0.91 (0.47, 1.77)
Moderate/severe AR/PVL	432/4043 (10.7%)	79/1314 (6.0%)	1.30 (0.99, 1.69)	1.30 (0.84, 2.00)
CAO	35/4441 (0.79%)	12/1410 (0.85%)	0.80 (0.39, 1.65)	0.80 (0.25, 2.59)
Valve dysfunction	136/4426 (3.1%)	40/1407 (2.8%)	0.78 (0.53, 1.15)	0.78 (0.41, 1.47)
Pacemaker implantation	520/4439 (11.7%)	130/1405 (9.3%)	1.30 (1.04, 1.62)^a^	1.30 (0.91, 1.86)
Device migration	72/4437 (1.6%)	24/1402 (1.7%)	1.21 (0.72, 2.03)	1.21 (0.52, 2.80)
Hemofiltration/dialysis	178/4426 (4.0%)	70/1405 (5.0%)	0.89 (0.65, 1.22)	0.89 (0.53, 1.48)
MVC	177/4431 (4.0%)	56/1407 (4.0%)	0.84 (0.60, 1.18)	0.84 (0.49, 1.45)
Early safety	1114/4386 (25.4%)	276/1391 (19.8%)	0.98 (0.83, 1.15)	0.98 (0.75, 1.27)

AR indicates aortic regurgitation; BAV, balloon aortic valvuloplasty; CAO, coronary artery obstruction; MI, myocardial infarction; MVC, major vascular complication; ORs, odds ratios; PS, propensity score; PVL, paravalvular leakage; TAVI, transcatheter aortic valve implantation.

a Significant at the 5% level.

**Table 3:** PS‐Adjusted ORs (After Bonferroni Correction) for Each of the Considered Outcomes in the Whole Cohort for the Sensitivity Analysis

Outcome	OR (95% CI) Before and During TAVI BAV (n=507)	OR (95% CI) Before and Not During TAVI BAV (n=197)	OR (95% CI) Not Before and During TAVI BAV (n=4466)
30‐day mortality	1.69 (0.79, 3.59)	1.01 (0.29, 3.49)	1.01 (0.59, 1.75)
MI	1.13 (0.12, 10.32)	NA	0.96 (0.22, 4.15)
Stroke	0.79 (0.24, 2.60)	0.57 (0.07, 4.80)	0.88 (0.42, 1.82)
Moderate/severe AR/PVL	1.60 (0.83, 3.07)	0.93 (0.30, 2.89)	1.28 (0.80, 2.06)
CAO	0.23 (0.01, 8.94)	0.61 (0.02, 23.89)	0.81 (0.22, 2.94)
Valve dysfunction	0.58 (0.19, 1.80)	0.54 (0.06, 4.54)	0.68 (0.34, 1.38)
Pacemaker implantation	0.97 (0.50, 1.87)	0.99 (0.39, 2.50)	1.28 (0.86, 1.89)
Device migration	1.54 (0.38, 6.23)	1.10 (0.13, 9.74)	1.16 (0.46, 2.91)
Dialysis	1.02 (0.41, 2.53)	0.98 (0.30, 3.23)	0.91 (0.52, 1.58)
MVC	0.66 (0.23, 1.90)	0.82 (0.19, 3.47)	0.83 (0.46, 1.50)
Early safety	0.97 (0.62, 1.52)	0.87 (0.43, 1.74)	0.94 (0.71, 1.26)

Note that the direct‐TAVI group was taken as the reference. AR indicates aortic regurgitation; BAV, balloon aortic valvuloplasty; CAO, coronary artery obstruction; MI, myocardial infarction; MVC, major vascular complication; NA, not applicable; ORs, odds ratios; PS, propensity score; PVL, paravalvular leakage; TAVI, transcatheter aortic valve implantation.

When studying patients treated with the balloon‐expandable SAPIEN valve, there were no significant differences in any of the endpoints between During‐TAVI BAV and Direct TAVI treatment groups (Table 4). For the self‐expanding CoreValve prosthesis, before multiplicity correction, the During‐TAVI BAV group had significantly lower odds of valve dysfunction (OR of 0.58) over those undergoing Direct TAVI. However, this finding was not significant after multiplicity correction (Table 4). Other endpoints were not significantly different across treatment groups in the CoreValve subgroup. Similar findings for the SAPIEN and CoreValve subgroups were observed in the sensitivity analysis of the 4‐treatment‐group analysis (Tables 5 and 6).

**Table 4:** Crude Event Rates and PS Regression Adjusted ORs for Each of the Considered Outcomes by Valve Type for the Main Analysis That Excluded BAVs Conducted Before TAVI

Outcome	SAPIEN Valve Patients (n=3201)				CoreValve Patients (n=2467)			
	During‐TAVI BAV (n=2336)	Direct TAVI (n=865)	PS Adjusted OR (95% CI) Without Bonferroni Correction	PS Adjusted OR (95% CI) With Bonferroni Correction	During‐TAVI BAV (n=1978)	Direct TAVI (n=489)	PS Adjusted OR (95% CI) Without Bonferroni Correction	PS Adjusted OR (95% CI) With Bonferroni Correction
30‐day mortality	137/2336 (5.9%)	40/865 (4.6%)	1.13 (0.76, 1.68)	1.13 (0.59, 2.15)	98/1978 (5.0%)	22/489 (4.5%)	0.80 (0.46, 1.38)	0.80 (0.33, 1.93)
MI	17/2322 (0.73%)	6/862 (0.70%)	0.73 (0.27, 2.02)	0.73 (0.14, 3.79)	17/1968 (0.86%)	1/482 (0.21%)	2.17 (0.26, 18.3)	2.17 (0.07, 68.61)
Stroke	55/2325 (2.4%)	23/860 (2.7%)	0.63 (0.37, 1.08)	0.63 (0.26, 1.51)	72/1969 (3.7%)	12/482 (2.5%)	0.97 (0.48, 1.95)	0.97 (0.31, 3.01)
Moderate/severe AR/PVL	121/2072 (5.8%)	32/800 (4.0%)	1.11 (0.72, 1.71)	1.11 (0.55, 2.23)	306/1830 (16.7%)	46/453 (10.2%)	1.15 (0.79, 1.68)	1.15 (0.63, 2.12)
CAO	19/2320 (0.82%)	7/860 (0.81%)	0.84 (0.32, 2.19)	0.84 (0.18, 3.97)	14/1970 (0.71%)	5/484 (1.0%)	0.41 (0.13, 1.35)	0.41 (0.06, 2.81)
Valve dysfunction	50/2315 (2.2%)	14/858 (1.6%)	1.12 (0.58, 2.16)	1.12 (0.39, 3.25)	83/1964 (4.2%)	25/483 (5.2%)	0.58 (0.34, 0.99)^a^	0.58 (0.24, 1.40)
PPM	127/2324 (5.5%)	45/858 (5.2%)	1.18 (0.80, 1.76)	1.18 (0.63, 2.24)	363/1967 (18.5%)	67/480 (14.0%)	1.26 (0.91, 1.74)	1.26 (0.74, 2.13)
Device migration	29/2325 (1.2%)	4/858 (0.47%)	2.65 (0.86, 8.14)	2.65 (0.43, 16.32)	40/1964 (2.0%)	18/481 (3.7%)	0.91 (0.45, 1.83)	0.91 (0.29, 2.83)
Hemofiltration/dialysis	116/2311 (5.0%)	44/859 (5.1%)	0.95 (0.64, 1.42)	0.95 (0.50, 1.82)	62/1966 (3.2%)	23/480 (4.8%)	0.91 (0.50, 1.65)	0.91 (0.35, 2.39)
MVC	97/2316 (4.2%)	31/857 (3.6%)	0.98 (0.62, 1.54)	0.98 (0.47, 2.04)	77/1966 (3.9%)	21/483 (4.3%)	0.79 (0.44, 1.40)	0.79 (0.31, 2.01)
Early safety	573/2282 (25.1%)	154/852 (18.1%)	1.07 (0.86, 1.34)	1.07 (0.75, 1.53)	521/1957 (26.6%)	111/473 (23.5%)	0.85 (0.65, 1.12)	0.85 (0.54, 1.32)

AR indicates aortic regurgitation; BAV, balloon aortic valvuloplasty; CAO, coronary artery obstruction; MI, myocardial infarction; MVC, major vascular complication; ORs, odds ratios; PPM, pacemaker implantation; PS, propensity score; PVL, paravalvular leakage; TAVI, transcatheter aortic valve implantation.

a Significant at the 5% level.

**Table 5:** PS‐Adjusted ORs (After Bonferroni Correction) for Each of the Considered Outcomes in the SAPIEN Subgroup for the Sensitivity Analysis

Outcome	OR (95% CI) Before and During TAVI BAV	OR (95% CI) Before and No During TAVI BAV	OR (95% CI) No Before and During TAVI BAV
30‐day mortality	1.71 (0.65, 4.44)	1.10 (0.27, 4.53)	1.08 (0.54, 2.17)
MI	0.84 (0.06, 11.85)	NA	0.70 (0.11, 4.23)
Stroke	0.84 (0.20, 3.50)	0.51 (0.04, 6.99)	0.61 (0.23, 1.58)
Moderate/severe AR/PVL	1.36 (0.47, 3.92)	1.15 (0.25, 5.32)	1.13 (0.53, 2.41)
CAO	NA	0.82 (0.02, 36.94)	0.83 (0.15, 4.59)
Valve dysfunction	0.95 (0.16, 5.73)	0.47 (0.01, 18.03)	0.99 (0.31, 3.12)
Pacemaker implantation	0.91 (0.28, 2.95)	1.43 (0.39, 5.20)	1.13 (0.57, 2.25)
Device migration	5.91 (0.53, 65.82)	NA	2.79 (0.38, 20.74)
Hemofiltration/dialysis	1.03 (0.34, 3.12)	1.23 (0.34, 4.50)	1.01 (0.50, 2.03)
MVC	0.49 (0.11, 2.29)	0.74 (0.11, 5.02)	0.94 (0.42, 2.09)
Early safety	1.01 (0.56, 1.83)	1.03 (0.45, 2.39)	1.03 (0.70, 1.52)

Note that the direct‐TAVI group was taken as the reference. AR indicates aortic regurgitation; BAV, balloon aortic valvuloplasty; CAO, coronary artery obstruction; MI, myocardial infarction; MVC, major vascular complication; NA, not applicable; ORs, odds ratios; PS, propensity score; PVL, paravalvular leakage; TAVI, transcatheter aortic valve implantation.

**Table 6:** PS‐Adjusted ORs (After Bonferroni Correction) for Each of the Considered Outcomes in the CoreValve Subgroup for the Sensitivity Analysis

Outcome	OR (95% CI) Before and During TAVI BAV	OR (95% CI) Before and No During TAVI BAV	OR (95% CI) No Before and During TAVI BAV
30‐day mortality	1.71 (0.46, 6.42)	0.84 (0.06, 12.13)	0.86 (0.33, 2.26)
MI	NA	NA	2.91 (0.06, 137.8)
Stroke	0.48 (0.05, 5.09)	NA	0.96 (0.28, 3.30)
Moderate/severe AR/PVL	1.90 (0.77, 4.65)	0.68 (0.12, 4.00)	1.13 (0.58, 2.17)
CAO	0.40 (0.01, 22.63)	NA	0.46 (0.06, 3.78)
Valve dysfunction	0.60 (0.14, 2.62)	0.64 (0.04, 9.23)	0.55 (0.21, 1.44)
Pacemaker implantation	1.14 (0.45, 2.86)	0.80 (0.18, 3.63)	1.26 (0.71, 2.23)
Device migration	1.21 (0.15, 10.03)	1.34 (0.12, 15.03)	0.89 (0.26, 3.06)
Hemofiltration/dialysis	0.84 (0.13, 5.51)	0.37 (0.01, 14.33)	0.88 (0.31, 2.53)
MVC	0.75 (0.14, 4.00)	1.43 (0.15, 13.91)	0.74 (0.27, 2.07)
Early safety	0.98 (0.47, 2.05)	0.69 (0.18, 2.61)	0.82 (0.51, 1.34)

Note that the direct‐TAVI group was taken as the reference. AR indicates aortic regurgitation; BAV, balloon aortic valvuloplasty; CAO, coronary artery obstruction; MI, myocardial infarction; MVC, major vascular complication; NA, not applicable; ORs, odds ratios; PS, propensity score; PVL, paravalvular leakage TAVI, transcatheter aortic valve implantation.

### Predictors of During‐TAVI BAV

**Figure 5:**
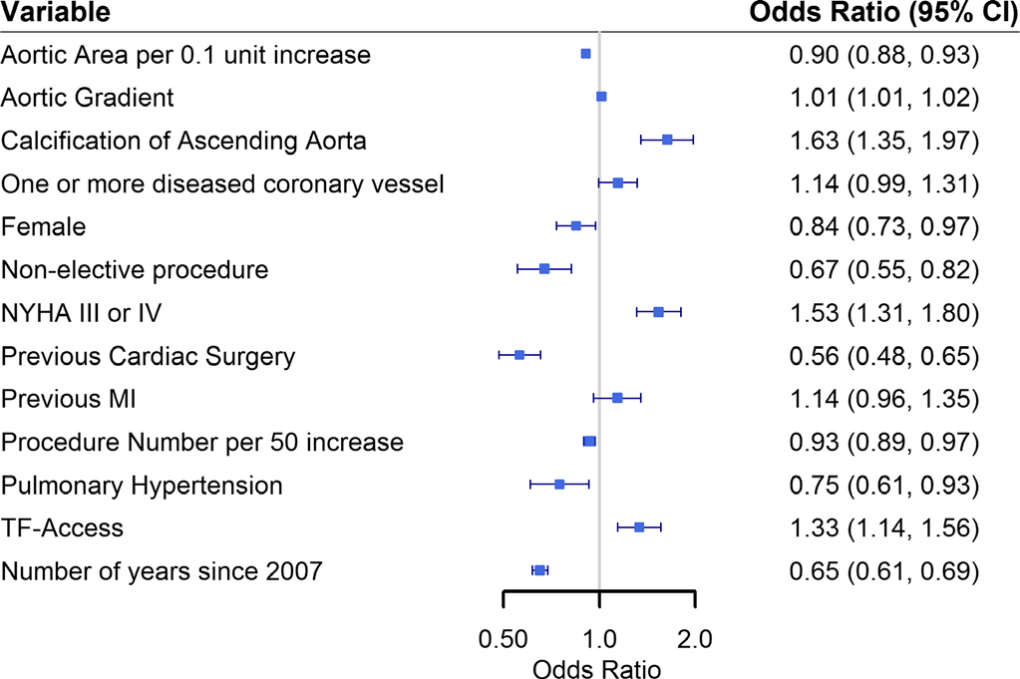
Odds ratios of variables that were identified as independent predictors of a patient being in the during‐TAVI BAV group. BAV indicates balloon aortic valvuloplasty; MI, myocardial infarction; NYHA, New York Heart Association; TF‐Access, transfemoral access route; TAVI, transcatheter aortic valve implantation.

Variables that were independently associated with the use of predilation are given in Figure 5. Odds of undergoing During‐TAVI BAV were significantly lower with increasing year of procedure and with increasing number of TAVI procedures for a given center, which supports the trend analysis after multivariable adjustment. Additionally, female patients with larger aortic valve area, previous cardiac surgery, pulmonary hypertension, and nonelective procedures were significantly less likely to undergo a During‐TAVI BAV. Conversely, calcification of ascending aorta, NYHA class III or IV, and transfemoral access were associated with significantly increased odds of During‐TAVI BAV.

## Discussion

This analysis of 5887 UK TAVI procedures has shown heterogeneity in the use of BAV nationally. Importantly, outcomes were not significantly different between patients who had a Direct TAVI and those who only had a BAV as part of the TAVI procedure. Notably, there were no significant differences in all outcomes across any of the treatment groups in SAPIEN valve patients. Similarly, after correction for multiple testing, there were no significant differences between those with and without BAV in patients treated with a CoreValve prosthesis. These findings support those from a recent meta‐analysis, which showed similar outcomes post‐TAVI both with and without predilation.^13^

Although using BAV pre‐TAVI may help to prepare the calcified aortic valve, stand‐alone BAV procedures are associated with several complications^6^, ^28^; hence, removing the predilation step may simplify the TAVI procedure. This study highlighted that the proportion of TAVI patients in the UK having a BAV in pre‐TAVI workup is decreasing through time. Despite predilation before TAVI valve deployment being the most common procedure throughout the majority of UK TAVI centers, several centers conducted relatively high proportions of Direct TAVI procedures. The reasons behind these changes in procedure are unclear from the current work, but certainly translate the progress along the learning curve that leads to more confidence with direct implantation.

### BAV Outcomes in SAPIEN Valve Patients

An important finding of the current study was that there were no significant differences over any of the clinical outcomes between treatment groups in the SAPIEN valve patients. These results are consistent with previous studies.^8^, ^9^, ^12^, ^14^, ^29^ A study that compared 50 transapical access patients with BAV to 50 transapical access patients without BAV found no significant differences in any of the endpoints defined in the VARC‐2 definitions^8^; this finding was later supported with studies on transfemoral access SAPIEN‐TAVI patients.^9^ In contrast, previous work has suggested that SAPIEN‐TAVI without BAV is associated with higher volume of cerebral ischemic lesions.^30^ In the current study, differences between stroke outcomes over the 2 treatment groups were not significant. Once published, findings from a planned multicenter 2‐armed observational trial (EASE‐IT) comparing SAPIEN TAVI patients with or without predilation will provide further insights.^31^ The present study suggests that SAPIEN TAVI procedures can feasibly be conducted without routine BAV, without increased risk in adverse outcomes. However, a degree of selection on a patient level is advocated, likely based on the extent of calcification and movement of leaflets, but also based on whether a patient has impaired LV function where one might want to minimize pacing time during TAVI.

### BAV Outcomes in CoreValve Patients

After correction for multiple testing, there were no significant differences with and without predilation in CoreValve patients. When testing many endpoints, one would expect to find positive results by chance simply attributed to the way hypothesis testing is conducted.^32^ Nonetheless, the feasibility of conducting TAVI without BAV in CoreValve patients was first proposed in a pilot study of 60 patients.^11^ Subsequent studies have shown that clinical outcomes are similar between BAV treatment groups in CoreValve patients.^10^, ^33^, ^34^ Theoretically, conducting TAVI without BAV in self‐expanding valves could potentially lead to worse outcomes. For example, without BAV, self‐expanding valves may not achieve as good expansion and may therefore fail to reach optimal deployment dimensions, particularly in heavily calcified aortic annuli. Whereas the current study highlights the potential to remove the predilation step in CoreValve TAVI procedures with regard to clinical outcomes, further work in this subgroup of patients will be required. For example, it is possible that patients undergoing CoreValve TAVI without previous BAV will require postdilatation more frequently to correct for stent under expansion and/or paravalvular leakage. The majority of patients in the current study did not have data on postdilation requirement, and so this endpoint could not be analyzed.

### Timing of BAV Relative to TAVI

We hypothesized a priori that the timing and indication for performing BAV could be related to the impact on subsequent clinical outcomes. Consequently, the sensitivity analysis included those patients who had a BAV as a bridge to TAVI (ie, a BAV completed before the date of the TAVI procedure), who represent a specific complex group of patients. All outcomes were similar between those who had a BAV before the date of TAVI (with or without subsequent BAV during TAVI) and those undergoing Direct TAVI. However, although we were able to distinguish the patients who had a BAV as a bridge to TAVI, the UK registry does not capture the reasons a BAV was conducted. Hence, this study could not investigate the full impact of BAVs conducted before TAVI. Before‐TAVI dilation is often conducted when a patient has presented with severe AS or when there are questions regarding the clinical benefit of a TAVI procedure. Therefore, one could argue that TAVI might not be feasible in such cases, without the period of convalescence after the preparatory BAV. Further work in such patients is recommended, given that there are a paucity of data for this specific cohort of patients.

### Limitations

One limitation of the current work is that outcomes associated with the decision to use BAV were studied in this retrospective study. Such a design may introduce significant selection biases given that the UK TAVI registry does not capture the reasons why or how each BAV was conducted. As such, any reported relationships cannot be interpreted as causal and they may relate to unmeasured confounders or selection bias. The inclusion of most patient demographic, procedural information, and TAVI center experience in the PS models should mitigate the effects of this as much as possible. Likewise, patients who undergo a BAV are generally more‐severe cases with complex anatomy and would hence be expected to have poorer outcomes over those who do not undergo BAV; the use of PS in the correct work aims to correct for such confounding by indication. Finally, the absence of information regarding hemodynamic performance, valve failure rates, and echocardiographic outcomes means that such outcomes were unable to be analyzed. Similarly, we were unable to investigate technical difficulties, which have previously been indicated in Direct TAVI patients.^14^

## Conclusion

This large‐scale study highlights that a no‐BAV (Direct TAVI) approach has similar clinical outcomes to the current practice of using BAV to predilate the diseased valve, especially when using a balloon‐expandable prosthesis. Although this analysis provides evidence that omitting the BAV step is feasible, this warrants prospective, randomized studies to define further the utility of BAV.

## Sources of Funding

This research was funded by the Medical Research Council, through the Health e‐Research Centre, University of Manchester (MR/K006665/1), and the North Staffordshire Heart Committee.

## Disclosures

None.
